# Faces of exclusion: the “social,” the “digital” and “digital racism” in a decolonial critical essay

**DOI:** 10.3389/fsoc.2025.1534313

**Published:** 2025-02-27

**Authors:** Lívia de Oliveira Mariano, Luciana de Santos Moura, Rodrigo Helder Paiani Mattos, Fabiana Pinto de Almeida Bizarria, Luciana Kind

**Affiliations:** ^1^Programa De Pós-Graduação Stricto Sensu em Psicologia, Pontifícia Universidade Católica de Minas Gerais, Belo Horizonte, Minas Gerais, Brazil; ^2^Faculdade De Psicologia, Pontifícia Universidade Católica de Minas Gerais, Belo Horizonte, Minas Gerais, Brazil; ^3^Professora Do Programa de Pós-graduação em Psicologia PUC-MG e Professora Colaboradora do Programa de Pós-Graduação em Gestão Pública, Mestrado Profissional da Universidade Federal do Piauí – PPGP/UFPI, Teresina, Piauí, Brazil

**Keywords:** decoloniality, digital exclusion, digital racism, subjectivation processes, memory

## Abstract

This article addressed digital racism and exclusion from the decolonial perspective, explicitly concerning the possibilities of resistance to colonial structures. This argument was based on the discussion of intersectionality as a reference to the multiple combinations of exclusionary experiences expressed here through new forms of hierarchizing difference, understood from the perspective of social classification, as taught by Aníbal Quijano. We theoretically rehearsed the topic to broaden dialogs, conducting a reflective exercise that invites debate. Based on the notions of subjectivation processes emerging from this elaboration, we performed a propositional reflection, pointing to plural and collective solutions that rescue the memory and knowledge denied by coloniality. Furthermore, we suggest the rediscovery of local practices and values, as opposed to the adherence to standards established by remnants of colonization that are transmitted and reverberated in contemporary daily life.

## 1 Introduction

In the contemporary social scenario, numerous forms of technology are intertwined with the lives of many individuals. Regarding communication technologies, including platforms and social media, it can be seen that they are interactive tools considered indispensable to life, playing essential roles in various everyday life spheres (Alves, 2017). Digital environments have thus become a stage where different social groups are formed. Discussions about complex themes are woven, having repercussions on the subjectivation processes of the people involved, as Neves and Portugal (2011) mention: “a subjectivity that is now constituted in the very act of making oneself visible, accessible to others,” which is produced “not only from the immediate interaction of humans among themselves but, mainly, from the interaction that we establish with the technical objects of our time” (Neves and Portugal, 2011, p. 21).

When we consider that Digital technology tools mobilize subjects in their daily lives, we also understand that there are repercussions on their relational and procedural subjectivities (Alves, 2017). Despite the almost omniscient and omnipresent presence of such tools, not everyone has access to them or knows how to manipulate them - a social phenomenon called digital exclusion (Moura et al., 2020), given the understanding that digital technologies reinforce racial, class and gender inequalities, producing new forms of racial hierarchy and differentiation (Animento, 2024). The recent history of political violence in Brazil has also seen the emergence of hate speech, which has been intensified by media connections (Duarte et al., 2018).

Sorj and Guedes (2005) indicate that the challenges of accessing the digital world have varied implications, such as unequal levels of education, geographic spaces, and gender issues. In this way, in a study developed in Indonesia, Karmila and Budimansyah (2022) observed acts of racism on digital platforms such as Facebook, Instagram, Twitter, TikTok, and YouTube, including hate messages, memes, videos, and expressions in live chats, which belittle others based on physical characteristics, generally associated with the idea of superiority of a specific race.

Aware of the problem, the United Nations Educational, Scientific and Cultural Organization (UNESCO) held the Global Forum against Racism and Discrimination on November 29 and December 1, 2023, in São Paulo. The event, which leaders from more than 20 countries attended, launched initiatives related to digital racism, among other topics and strategies. During the Forum, a report was launched that places regional data on racial discrimination in the virtual environment - the UNESCO Global Panorama against Racism and Discrimination Organização das Nações Unidas, (2023).

Still, on the national scene, the Federation of Industries of the State of São Paulo (Federação das Indústrias do Estado de São Paulo - Fiesp) and the Zumbi dos Palmares University promoted, in October 2023, a *hackathon*,^1^ with the theme “Deconstructing Racism in Digital Relationships.” Developers, programmers, designers, communication, marketing and sales professionals faced the challenge of proposing projects to “deconstruct the aggression and racial intolerance manifested on social media, e-commerce, distance learning and other relationship channels mediated by technological solutions” (Junior, 2023, para. 3).

Delving into the understanding of the phenomenon, based on Ferreira (2020), it is understood that Digital platforms often reflect and amplify existing racial biases, while the architecture of these technologies embodies racial logic, perpetuating the marginalization of non-Western identities and experiences. In this context, digital exclusion manifests as a “digital apartheid,” characterized by the exclusion of large population groups from access to information and communication technologies. The matter is presented by Ferreira (2020), who situates the discussion of the exclusion cycle that prevents black students’ full participation in learning processes. With this, it is understood that power structures reduce democratic participation in these spaces by groups considered minorities (Galpin, 2022).

Mendoza (2022), when presenting the work by Graham and Dittus (2021), states that although the perception that the internet is a democratic space, a neutral space, there are significant barriers that limit full participation, such as digital literacy, access to formal education and the availability of free time, a topic also discussed by Moura et al. (2020). Inequalities result in a digital exclusion that disproportionately affects marginalized groups. At the same time, non-neutral or impartial technology (Benjamin, 2019) emerges as a tool for perpetuating structural racism since participating communities tend to disseminate dominant patterns in universalizing digital representation with territories and social groups.

Before the challenges that digital culture imposes regarding the idea of racism and exclusion, Bonhomme and Alfaro (2022) present the definition of “online cultural racism” as a form of “covert” racism that emphasizes the need for control through the construction of an inferior other, without resorting to dehumanizing racist discourses. On the other hand, there is “online aggressive racism,” referring to more radical and aggressive forms of hate speech, which use dehumanizing metaphors and expressions of explicit hatred against groups, and this form of racism is often more evident. Based on the idea of Alves (2017), who suggests a continuous relationship between the *online* and *offline* environments, we situate the internet as a space for interaction and formation of public opinion, which amplifies *offline* positions in a continuous relationship with the *online* environment. Thus, it allows racist speeches to go viral and influence social perception and even political direction.

Considering the discussion raised, digital racism and exclusion are put up for debate from the decolonial field, specifically about the possibilities of resistance to colonial structures, since in the digital space, even if we identify forms of exclusion, counter-narratives and digital guerrilla tactics can dispute the online occupation (Franco, 2022; Zafra, 2019). Thus, based on the notions of subjectivation processes emerging from this debate, we performed a propositional reflection given the possibilities of interaction that are more welcoming to difference.^2^ Such debate relies on the discussion of intersectionality as a reference to the multiple combinations of exclusionary experiences expressed in this discussion based on new forms of hierarchizing difference, understanding it from the perspective of social classification, as Aníbal Quijano teaches. It is important to stress that some of the references used are dated, especially those that discuss digital racism and digital exclusion – which occurs due to the limited production found in this filed, highlighting the importance of this paper.

To follow the argument, we seek a methodology that allows discussion on the topic and expands dialogs in theoretically rehearsing a theme, as Marsal et al. (2014) advised. To this end, the authors must place themselves in the text and position themselves concerning the proposed theme based on arguments (Marsal et al., 2014). From this perspective, we also consider it essential to elucidate the sociocultural dimension of these positions – Brazilians, therefore, mobilized by the historical dimension of subalternity inscribed in the Brazilian “being,” while “the essay requires subjects, essayist and reader, capable of assessing that understanding reality also occurs in other ways” (Meneghetti, 2011, p. 321), including “their efforts still reflect the availability of someone who, like a child, is not ashamed to be enthusiastic about what others have already done” (Adorno, 2003, p. 16).

The essay, then, does not intend to be a closed construction. Therefore, it is assumed to be an unfinished, essayistic process, a reflective exercise and an invitation to reflection and debate in the search for a better understanding of phenomena that present themselves to the authors, disturb them and lead to elaborations on emerging issues (Adorno, 2003) - digital exclusion and racism, while being objects of analysis, are also understood as phenomena arising from and sustained by the phenomenon of coloniality, in other words, that which presents itself to the authors, and which worries them (Meneghetti, 2011).

## 2 Getting started: the decolonial field

The decolonial field is defined as the field of studies that reflects on the context of colonization experienced by several nations, generally associated with the global South,^3^ that problematizes the structures of domination and exploitation remaining from this period (Quintero et al., 2019), reverberating to the present day, in the context of values, beliefs and social practices, in what Quijano (2009) defines as the coloniality of power, knowledge and being. From power, which is configured by the axis of the exploitation/domination/conflict relationship, when intersections of work, race and gender represent effective means of maintaining control of power by the capitalist order and therefore reproduce tools for maintaining asymmetrical power relations; from knowledge, related to the production and validation of knowledge, as a driving force of capitalism; and from being, which, according to Quijano (2009), is related to the way people define themselves, construct understandings about their existence, and interact to the world, naturalizing social relations based on hierarchies associated with differences.

Considering the criticism of the development of patterns associated with the global North, disseminated and legitimized based on the colonial relationship (center-periphery), we understand the phenomena of exclusion and violence, which occur against certain groups situated on the margins of society, before justifications valued around economic development, supported by the civilizing representation of a liberal society, in a progressive conception that provides scientific evidence for the exclusion of various possibilities of being and existing in society (Lander, 2005). As former colonies, the countries and their native peoples had their own beliefs, cultures, and ways of being silenced so that, in their place, they could be considered inferior, and Eurocentric ideals (civility, progress, development) could be installed.

The denial of colonial knowledge, justified by the modernity rhetoric (Alatas, 2003), explains epistemic violence, or epistemicide, which implies the deliberate destruction of the people’s knowledge and culture, of others, of the uncivilized. As an idea of salvation, the civilizing conception supports epistemologically formative narratives of the Northern pattern of social life (Lee et al., 2015) that legitimizes the occupation of territories and the exploitation of indigenous peoples, as discussed by Pehuen et al. (2019).

The social classification process presented by Quijano (2009) is the central aspect of the analysis proposed around the decolonial field, understood in the horizon of division, separation and hierarchization of human beings based on markers of gender, race, ethnicity and social class. A process resulting from the colonial logic that associates colonized peoples with a social reading of inferiority (uncivilized), stigmatizing their existences in the field of invisibility, faced with denial, destruction and oppression - disseminated in the understanding of the power coloniality, which legitimizes; from knowledge, which imprints understanding; and from being, which mobilizes existence patterns under civilizing agenda of economic development. Thus, practices and their agents are excluded by a civilizing process, justified by the “expansion of a democratic political culture” (Dussel, 2016, p. 60).

From the perspective of coloniality, it also situates insights into racism about the relationship between exploitation and domination in the sphere of corporeality, understood as decisive in power relations, whereas “[in] exploitation it is the body that is used and consumed in work and, in most of the world, in poverty, hunger and malnutrition, in disease, punishment, repression, torture and massacres during the struggles against the exploiters” (Quijano, 2009, p. 113).

This discussion can be presented with the definition of “abyssal thinking,” which, in radical terms, represents the division of social reality into two distinct universes, understanding that there is another side of the line that does not exist as a reality - where, in this case, the global South is located.

… the experiences, identities and historical relations of coloniality and the geocultural distribution of global capitalist power were also formally *naturalized*. This mode of knowledge was, by its nature and origin, Eurocentric. Termed rational, it was imposed and adopted throughout the capitalist world as the only valid rationality and an emblem of modernity. The guiding principles of this cognitive perspective have remained, despite changes in their specific content, criticism and debates, throughout the global power of colonial and modern capitalism. This is the modernity/rationality that is now, finally, in crisis (Quijano, 2009, p. 74).

Around this division, there are also understandings about universal binary categories, such as “true” and “false,” “man” and “woman,” “development” and “underdeveloped,” “scientific knowledge” and “non-scientific knowledge,” including associating the “non-scientific” with incomprehensible, magical and idolatrous practices, or a “non-knowledge.” In this sense, there is a denial of human nature itself, which reverberates in different ways in which the subject perceives himself, perceives others and perceives the world around him, including about superiority (men, white people, heterosexuals, among others) and others as inferior (women, non-white people, LGBTQIA+ people,^4^ between others).

As Quijano (2009, p. 105) points out, “in power relations, certain attributes of the species played a central role in the social classification of people: sex, age and workforce […] from America the phenotype was added.” thus, around the reading of “being,” from the perspective of the decolonial field, the coloniality of being is discussed, considering social and historical references that affirm the existence of being as subjective processes, as a hidden agenda of modernity (Lander, 2005).

## 3 Next steps: from the being coloniality to the subjectivation processes

In dialog with Mignolo (2003) and Maldonado-Torres (2008) argues that the relationship between power and knowledge leads to the conception of being, while science cannot be separated from languages and, as a *loci* of enunciation, knowledge, historical-social-cultural being is inscribed, which situates the formation of identities, procedurally, in interaction (acceptance/denial; inclusion/exclusion).

Processuality, in light of epistemic violence, suggests that the production of existences in the subjective fabric marked by experiences of social exclusion places a being in dramatic situations of non-existence, heightening tensions facing the affirmation of being in narratives of invisibility, denial, oppression – understood as conflicts of (mis)adaptation to narratives, to discourses that classify and define the possibilities of existence. Otherwise, the processuality of lives and experiences understood in light of colonialities supposes that identities, as identification, tension (conflicts) the existence of the being with the normative standards of colonial processes (the power and knowledge coloniality), which deny (via discursive constructions) possibilities of existence (in narratives formation) in coherence logic (adaptation, misadaptation) of the values that sustain modernity, related to the capitalist social system.

Furthermore, regarding procedurality, we reflect on intersectionality, inspired by Collins et al. (2021), aiming to understand the varied experiences of exclusion due to social classifications that hierarchize differences due to universal social standards. The term intersectionality radicalizes the drama of conflicts of (mis)adaptation confronted with inclusion–exclusion based on layers, markers that make existence vulnerable. Understanding intersectionality according to social classification in the decolonial field invites us to comprehend epistemicide as material, physical and symbolic destruction that imprints on the coloniality of being the experience of colonial suffering – the denial of existence.

The subordination of practices and subjectivities of dominated peoples before the understanding of epistemic violence that puts tension on(mis)adaptation to narratives, to discourses that classify and define the possibilities of existence, in ways of being and living inscribed in the universalization of the societal and cultural form, originating from Eurocentric standards (Spivak, 2010). The suffering that speaks of wisdom *uprooting*. As Parra-Valencia and Galindo (2019) cite, in the death from the knowledge of historically subalternate peoples associated with native peoples and racial conditions, white skin begins to be considered superior to yellow and black skin in terms of psychological, moral, intellectual and cultural qualities.

According to Parra-Valencia and Galindo (2019), referring to Spivak (2010), epistemic violence operates in everyday practices of recording and memory and subjectivation processes. From a memory that “cannot” be named, signified, felt or elaborated (narrative coherence to discourse) because it has been denied, placed on the margins of human experience. In this way, affirming the decolonial field^5^ suggests enabling the recall of overshadowed, denied experiences and rescuing the silences as a subalternate people as “remnants of colonization that are transmitted and that reverberate as body marks of colonization, and that return contemporaneously in the body” (Cárdenas, 2023, p. 23).

Epistemic violence also produces suffering as a result of the tensions of the experience of being, a representation that subjects have of themselves permeated by forgetfulness, silence and fear, by the exclusion resulting from multiple (in)existences. As Quintero et al. (2019, p. 5) point out, “the power relations asymmetry between Europe and its others represents a constitutive dimension of modernity and, therefore, necessarily implies the subordination of the practices and subjectivities of dominated peoples.”

Considering reflections derived from Gómez (2015) and Quintero et al. (2019), memory social inscribes silenced knowledge in language, directing the repositioning of “Selfs” in various centers before the (re)cognition of other codes through our experiences that would enable other interpretations, other social senses and, consequently, other meanings – reconnection to the social, affective, ethical, aesthetic worlds, among others. The (r)existence to the fragmentation of reality and being, catchable to the compression of the power, knowledge and being coloniality, in rescues of reminiscences of our social history supported by reflective seams –in the possibility of other narratives of (r)existence on the horizon of colonial discourses matrix.

As a process of a being that, subjectively and historically, situates its existence in perspectives of coloniality, subjectivity is addressed in the singularization process of human experience, recognizing the complexity and historicity of subjects, as defined by Prado Filho and Martins (2007). Inspired by Michel Foucault, the authors problematize the psychological discourses that delimit the object of Psychology studies as a field of the subject’s experiences. Psychologies are called to reflect on the practices of subjectivation and power relations in favor of dismantling the naturalizations of the psychological. The authors defend a psychology decentered from the subject because “if the subjectivation modes subjectify, singularization presents itself as an aestheticization of the self aiming to resist this modern machinery of production of individual subjectivity and identity, constructing new forms of life and being” (Prado Filho and Martins, 2007, p. 18).

Leite and Dimenstein (2002), following the same path as Prado Filho and Martins (2007), considering contributions from Michel Foucault, Gilles Deleuze and Félix Guattari, overcome the traditional view of subjectivity that considers it an individualized, universal, structural and rational experience, assumed since modern science, reducing the subject to an isolated entity. Therefore, they recognize an understanding of subjectivity more closely articulating social processes and interactions, historical contexts, and power relations.

As Prado Filho and Martins (2007, p. 10, emphasis added) point out, the idea of the production of subjectivity is based on a processual dimension, with attention to historicity.

… as an alternative to a problematization of ‘identity’ *precisely because it seeks to account for differences.* This historical-political perspective of subjectivity gains prominence at this time due to the decline of the concept of identity, which is exhausted in an exaltation of the ‘identical’: this movement of repeating oneself, of making oneself identical to oneself *to facilitate social visibility and allow the localization and capture of powers*. Visibility is in two ways: from the subject who repeats himself and recognizes himself as identical to himself and who, in this movement, exposes himself to the view of others, *becoming identifiable and captureable by law, norms, and morality*. Therefore, this political issue is linked *to practices of individualization and social identification of subjects, involving games of normalization, forms of recognition of oneself and others,* besides modes of subjectivation, *which require critical positions and resistance* to particular ‘identities politics’ exercised by the contemporary State.

Considering the reflections raised by Leite and Dimenstein (2002) and Prado Filho and Martins (2007), identity is understood as the result of tensions in experiences linked to coloniality, in the desire for existence in the presence of denial referenced by epistemic violence. Among the processes of subjectivation is the exercise of identity, as identification and social recognition, in the experimentation of one’s own social experience within the horizon of societal values associated with citizenship, in the field of human and social rights – from a universalizing movement, the rules of social groups that circumscribe identity-identification are defined, while subjectivation processes.

When delving into the concept of identity, Ciampa (2012) defines it by considering the asymmetries of power present in social relations, assuming the processuality as constructed from the social and historical experiences that support “new’ narratives for the subjective construction of being. He also understands “identity as a contradictory, multiple and changeable totality, *yet one*. No matter how contradictory, no matter how changeable it may be, I know that I am the one who is like this, that is, *I am a unity of opposites, I am one in multiplicity and change”* (Ciampa, 2012, p. 61, emphasis added). The identity that presents itself as unity amid experiences that demand unity and coherence, based on social classifications that define it, confers the existence of social discourses that legitimize and inscribe ways of being and acting. From the exaltation to the identical, as Prado Filho and Martins (2007) cite, we understand the pressures for the law, the norm, and morality arising from colonialities in exercises of affirmation and denial of existence in narratives favorable to unity. In this reflection, the power of diversity and change from the opposites is suffocated and denied by discourses on the power-to-be.

The unity, identity-identification, resulting from subjectivation processes in the wake of the decolonial field are understood to be supported by the logic of coloniality, integrating into the subjective experience meanings coherent with universalizing discourses in hierarchically defined social classifications, with dissonant experiences being denied and forgotten, as cited by Gómez (2015) and Quintero et al. (2019). This aspect raises the possibilities of social existence in the context of a society that recognizes the being based on their social identities, including in terms of validating their existence as citizens, guaranteeing their rights – “to be,” “to know,” and “to be able to be,” standardized through the conservation of produced identities and the repositioning of assumed identities, contributing to the maintenance of social structures. Then, identities result from subjectivation processes defined by the social, political, and cultural contexts and required before social existence. They are anchored by the political dimension that gives visibility to being grounded on their identifications. In this way, we observe in Ciampa (2002, p. 1).

The issue of group identity politics involves the discussion about autonomy (or not), which transforms for individuals into questions about the authenticity (or not) of political identities, perhaps reflecting two opposing views, depending on whether the emphasis is on equality – a society centered on the State – or on freedom – a society composed of individuals (Ciampa, 2002, p. 1).

Supported by the decolonial debate, the understanding of identity policies reflected by Ciampa (2002) is expanded when referred to as subjectivation processes mediated by power relations (power coloniality), the knowledge that sustains (knowledge coloniality) and validations of ways of being (being coloniality). Thus, they suggest tensions related to the search for consensus emerging from conflicts, announced by Ciampa (2002) as a dialectic between progress/development and oppression/exploitation, in the context of democratic projects that require an articulation between the construction and recognition of new identities with the legal self-organization of free and equal citizens.

Inspired by Habermas (1997), in his contributions to Critical Theory in the discussion of communicative rationality, Ciampa (2002) announces the possibility of problematizing the understanding of identities with support in dialogical processes that mobilize transformations on the path to emancipation. From this point, he questions the narratives capable of resisting discourses anchored by social classifications that demand identities-identifications in the context of affirming existence-citizenship-rights.

Colonial power dominated territories and peoples and shaped colonized people’s identities (Quijano, 2005). Resistance, announced as an emancipatory possibility, is central to Ciampa (2002) and the decolonial field. What is discussed below is that epistemic violence contributes significant challenges to the (re)cognition of the plurality of ways of being and living – in which identity-identification as a framework configures and restricts, through narratives inscribed in discourses about the “power to be” to be and exist – to adapt, to organize itself. The denials, forgetfulness, and oppression of the process may reflect the suffering of possibilities placed on the margins, a problem associated with social exclusion. In contrast, the perpetuation of racial, social and cultural hierarchies that resulted in oppression and inequality are still present in contemporary times (Fonseca, 2021). In this sense, Quijano (2009, p. 109) is taken up again.

In the center (Eurocenter), the wage relationship was the form of the capital-labor relationship structurally and demographically dominant in the long term. In other words, the wage relationship was mainly “white”. On the other hand, in the “colonial periphery”, the wage relationship was structurally dominant over time but always a minority demographically as elsewhere, while the most widespread and sectorally dominant were all the other forms of labor exploitation: slavery, servitude, simple commodity production, reciprocity. But all of them were articulated under the rule of capital and for its benefit.

Understanding the subjectivation processes that reference identities-identifications under coloniality supports the debate on the social exclusion processes endorsed by the social dynamics that inscribe, via hierarchical social classifications, the narratives of existence in coherence with the social discourses of modern origin in the field of values that sustain the economic dynamics of capitalist development. From the possibilities of exclusionary experiences, that which defines itself in the digital field draws the most attention as a debate in this essay, given the potential for dissemination associated with social networks, with enhanced repercussions and the difficult configuration of the problem and its consequences for those involved.

For Becker et al. (2022), the dynamic, fluid, accelerated and immediate logic of contemporary times causes various social situations to undergo a reconfiguration in their limits, “allowing people to immediately fulfill various social roles at all times, which impact the associated subjectivation processes” (Becker et al., 2022, p. 110).

Such reflection is presented as a critical decolonial exercise, understanding that it is fundamental for psychology to understand the problem from various lenses – those that mobilize political action before identities-identifications that (r)exist in the discourses of universalization of existence and their recognized social groupings; those that assume identities-identifications as requirements of existence, also conformed to the logic of totalizing discourses, enhancing the exercise of identification coherence that imprints the denial of difference in identity confronted with the demands of identity policies. As a result, “the control of the production of resources for social survival and the control of biological reproduction, of the species” (Quijano, 2009, p. 101), that is, the control of life as an argument that supports the system’s mode of reproduction, the control of pleasure and descendants, including as preservation of property. Vivian Santos (2018, p. 4, emphasis added) reveals:

Coloniality, as the *permanence* of the colonial power structure, has as its main foundations: ‘racialization’ and the intrinsic racialized forms of production relations; ‘eurocentrism’, as a form of production and control of subjectivities, of existences; the hegemony of the ‘nation-State’ which, as an intrinsic process, after colonialism, is constructed as a periphery. Thus, *the colonial enterprise remains alive* through these foundations, materializing itself as a coloniality of power, knowledge and being.

The sufferings resulting from both paths are understood as triggered by the plots of the subjectivation processes understood by way of the decolonial field – meanings that define, through social and historical processes, senses of existence in coherence with the dynamics of values that support narratives about the possible ways of life for the discourses that define identity politics, whose social dynamics reflect the centrality of the capitalist model (Quijano, 2009). A vision of the world and life in demarcations, splits and dualisms – divisions that are also defended as essential for science (body *versus* mind; objective *versus* subjective, etc.), a classificatory condition of Being, for example, work, race and gender – the latter, worked emphatically by Lugones (2014).

## 4 The plots of the subjectivation process around digital racism

Digital platforms are regulated in Brazil by Law 14,532 of January 11, 2023, (Brazilian Presidency of the Republic, 2023) which prohibits racist practices by classifying them as crimes of racism and racial abuse, with a penalty of two to five years in prison. Although the regulation by the legislative branch is a step forward, we emphasize that Law 14,532 is recent and the use of social media is widespread, which means that challenges persist, considering ideas associated with freedom of expression and, consequently, impunity, given that the digital environment allows, to a certain extent, anonymity.

The concept of digital racism seems to be recent, with most scientific productions on the theme dating within the last 10 years – for example, the work of Roshani (2016), who uses the term despite not focusing on it. More recent productions, however, concentrate on the discussion of digital racism, including using the concept on their titles, such as the works of Ozduzen et al. (2021), Siapera and Viejo-Otero (2021), Agudelo and Olbrych (2022), and Karmila and Budimansyah (2022).

According to Ekman (2024, p. 2), digital racism is represented by “racist content produced and circulated online” and there are several forms and expressions of racism in this environment. From the same perspective, Siapera (2019) questions whether there are differences between offline and online racism and emphasizes the contrasts of an everyday and “banal” digital racism and the type that is organized and extreme, often associated with the far right. In dialog with Lentin (2016), the author also discusses two kinds of racism: a frozen one, which is intelligible and seen as unacceptable, and a motile one, that “becomes a moving target, the subject of discussion, denial and questioning” (Siapera, 2019, p. 3), often experienced as micro-aggressions.

Similarly, social media corporations have developed their own terms of service (Twitter), or ‘community standards’ (Facebook), which draw upon these understandings of illegal hate speech, but which also allow certain contents to circulate under rules governing freedom of expression and public debate. In this manner, we obtain a dichotomy of what effectively constitutes ‘acceptable racism’ and what is deemed unacceptable or even illegal (Siapera, 2019, p. 3).

An example is the platform X, formerly named as Twitter. The billionaire Elon Musk purchased X (then, still Twitter) in October 2022 for $44 billion and fired top executives and other employees of the company. Since then, several controversies involving Musk and X have been reported by news channels, such as the suspension of the social media in Brazil (BBC News Brasil, 2022). After Musk purchased X, tensions between the billionaire and the Minister of the Federal Supreme Court of Brazil began to grow. The company disobeyed several orders from Brazilian Justice Department, including removing profiles that published content that was pro-coup and/or attacked the country’s institutions (Bataier, 2024).

These orders are linked to the last presidential election in Brazil. After Luiz Inácio Lula da Silva’s victory in October 2022, supporters of former far right president Jair Bolsonaro, dissatisfied with the result and instigated by theories about fraud in the electoral process, attacked, on January 8, 2023, the National Congress, Planalto’s Palace and the Federal Supreme Court in Brasília. Following the attacks, the Supreme Court’s minister began to order the suspension of accounts that were allegedly linked to the attack and the dissemination of fake news (Bataier, 2024; Hising, 2024). After not complying with the requirements, the social media announced the closure of its office in Brazil on August 17, which led to a suspension of X in the country for 39 days, until October 9 – when the orders were carried out (CNN Brasil, 2024).

This case highlights how racism in the online environment also comes from the owners of the companies that run these platforms. Elon Musk is a white man who, despite being born on the African continent, is a naturalized American and Canadian, and is frequently related to the right. The tech billionaire, who is to co-lead the Department of Government Efficiency of the United States named by Donald Trump, often takes a prejudiced stance with racist (Valinsky, 2023), sexist (Spangler, 2024) comments, among others, with the justification of “free speech.” Williams (2024, para. 13), on an article published on “The Guardian,” states that “Musk’s commitment to free speech is jaw-droppingly unconvincing: he used it to reject Brazil’s demands, yet readily acceded to Narendra Modi’s demands in India, and suspended hundreds of accounts linked to farmers’ protests there in February this year.”

It is clear that, by using his money and power to accomplish his political interests, the CEO is reaffirming his pact with whiteness, i.e., a racial identity and a dimension of social structure that classifies whites as the dominant group, offering privileges to them (Bento, 2002; McDermott and Ferguson, 2022). In another words, as said by psychologist and activist Maria Aparecida Bento while discussing whiteness in Brazil, there is a tacit agreement among whites: whilst they do not recognize themselves as an essential part of the persistence of racial inequalities, they can maintain their *status quo*. McDermott and Ferguson (2022, p. 259) claim that “When power is not recognized, opposition is difficult to assemble. For this reason, rendering the subtle manifestations of white privilege and white supremacy apparent has been a main task of sociologists of whiteness.”

Thus, by failing to enforce regulations that could decrease hate speech under the guise of “free speech,” Musk and others are creating an environment propitious to the proliferation of digital racism – and, sometimes, even practicing digital racism themselves. Even though the suspension on Brazil is not digital racism *per se*, it highlights how the system can support the practice of hate speech against minorities.

As cited, the widespread dissemination of discourses associated with digital racism involves both organized supremacist groups and ordinary users, which poses challenges to tracking and analyzing their repercussions (Siapera and Viejo-Otero, 2021). Ozduzen et al. (2021) argue that the ideal of freedom of expression spreads hate speech in digital spaces.

From the problems understood in the matrix of modern thought, with greater attention on the coloniality of Being (Maldonado-Torres, 2008), it is clear that power and knowledge lead to the definition of Being. In the debate on subjectivation processes that support identities-identifications, it is argued that social classification, as defined by Quijano (2009), references subjectivity, from the notion of the subject’s space in society, in attention to the relations of domination and subordination between groups, for example, based on racial criteria, which justify the exploitation and marginalization of certain groups. Consequently, it can increase violence and racism, which result in the suffering of various kinds in the subjects’ lives (Parra-Valencia and Galindo, 2019).

We, the essayist authors, start from concerns in our daily lives. We observe racist comments in various posts through the use of social media despite policies that seek to curb hate speech. As observed in the research by Agudelo and Olbrych (2022), racist expressions exist as a mechanism that maintains the structures of oppression and racial privileges, which are understood as “coherent” with coloniality dynamics.

In this sense, it is important to situate where we are coming from: Latin America, Brazil, a former Portuguese colony. This work, besides being a research, is a part of our process of (re)encountering ourselves, our history and our memory through the reflection that decolonial thinking enhances. Thus, by producing knowledge that defies the white, European, north-centered norm, we are also bringing the problem to the light – which is key to assemble an opposition, as taught by McDermott and Ferguson (2022) – and questioning the structures left by colonialism.

Daily life also highlights, through news in major newspapers, the problem of digital racism. Through the series *United Shades of America*^6^, CNN in the United States announces the idea of a “war” on civil rights, thinking not only about the physical occupations of the streets but also those that occur on virtual and algorithmic routes in digital spaces. Besides, Siqueira (2019) states that inequalities, as an indistinct feature of modernity, are present in digital spaces based on the control of technological power exercised by a few (Siqueira, 2019). Digital exclusion, in line with digital racism, can be seen in two directions. The first is exclusion due to racist discourse, which can make the subject want to leave the *online* environment voluntarily. The second is exclusion due to a lack of access, either because of a lack of tools knowledge or difficulties arising from geographical and/or social issues (Mari, 2020). These two directions are generally associated with marginalized populations, social groups defined as social “minorities” marked by experiences of intersectional exclusion.

Agudelo and Olbrych (2022), in line with Bonhomme and Alfaro (2022), show that racism in the digital environment can be a discursive and digital form of discrimination, often presented as more subtle and polite without prominent racist language on social media. The reference of understanding whether or not the discourse is violent, in turn, inspires reflection, while acceptability is announced as an essential problem for the decolonial field.

Although SMP [*Social Media Platforms*] like Twitter have implemented different usage policies to control racial hate speech on their site, this still leaves the door open for *negotiations on what constitutes acceptable racial conversations and what constitutes racist speech.* Racist expressions on social media are not always easy to identify and can be less than obvious (Agudelo and Olbrych, 2022, p. 7, emphasis added).

The difficulty in identifying such discourses results in “flexible” racism, that is, via *online* policies, decontextualized to locate and understand racist practices, giving rise to the understanding that racism is, in a certain way, permitted (Siapera and Viejo-Otero, 2021; Agudelo and Olbrych, 2022). The circulation of this form of racism is orchestrated in “coherent” narratives with discourses triggered by social classifications that imprint on identity-identification the possibilities of existence, including denial of difference. This difficult demarcation and recognition makes the people involved vulnerable, increasing the suffering of those excluded and violated.

It is noted that this hidden condition makes it difficult for people to recognize violence since the condition of colonialities naturalizes violent experiences. There are often justifications for violence in the relations of domination, inscribed in the ways of being and acting, incorporated as legitimate even by the victim, who is equally entangled in the coloniality of being. The recognition of violence is presented from the perspective of decolonial criticism, which is also a field that is tensioned by social existence anchored by identity-identification, resulting in disputes over power, being able to be, that potentially deny the plurality of ways of being within identities-identifications – resulting in conflicts among and within social groups.

Digital racism, from this perspective, is characterized by violent discourses widely disseminated in the *online* environment (Agudelo and Olbrych, 2022), which are generally directed at people historically marginalized or “ethnic minorities,” as defined by Pérez et al. (2007). The difference, resulting from classification and hierarchy, as an “ontologization” situates readings that justify discrimination and violence when, for example, a minority is associated with non-human characteristics, dehumanizing the social group of reference (Pérez et al., 2007).

It is understood that psychology, as a science and profession, considering readings from the decolonial field, could reflect on the subjectivation processes of reference to identity-identification in the course of narratives inscribed in social discourses sustained by the dynamics of coloniality to understand the phenomenon of social and digital exclusion before digital racism. It becomes imperative to exercise reflection on the subjective plots triggered by the social classification that sustains, since modern logic, hierarchies, which mobilize understandings about “differences” in logics of exclusion, as epistemic violence.

In this sense, the recovery of memories, affections, values and collective notions can represent a counterpoint to the individualistic logic and supposed equality in terms of citizenship and human rights that are debated by the idea of identity-identification in the logic of identity politics, with implications, including, for the ideas of justice and democracy, as referenced by Gómez (2015). Above all, to discuss the subjective consequences of this process for the subjects involved and to foster spaces that make possible understandings about identities-identifications before the affirmation of being. For this, Psychology anchored in the possibilities of confronting digital racism is necessary.

Plural, collective solutions that rescue memory, that (re)know knowledge denied by coloniality and that (re)conceive fragmented subjectivities (Gómez, 2015) are a path that we consider possible, including Psychology as a science and profession, socially committed to being, (re)knowing the sufferings arising from the new exclusionary social phenomena. Furthermore, rediscovering local practices and values, as opposed to adhering to standards set by people who do not experience our reality and know little about it.

It is essential to highlight the intersectional dynamics involved in these processes. The concept of intersectionality follows the logic of recognizing discrimination, exclusion and violence suffered by minority groups. According to Crenshaw (2002), the conception of intersectionality seeks to capture the structural and dynamic effects of the interaction of multiple axes of subordination, that is, to conceptualize how discriminatory systems, when associated, result in fundamental inequalities that structure the relative positions of subjects.

Using an intersection metaphor, we will first draw an analogy in which the various axes of power, such as race, ethnicity, gender, and class, constitute the avenues that structure the social, economic, and political terrains. It is through these avenues that the dynamics of disempowerment move. These avenues are sometimes defined as distinct and mutually exclusive axes of power; racism, for example, is different from patriarchy, which, in turn, is distinct from class oppression. Such systems often overlap and intersect, creating complex intersections in which two, three, or four axes intersect (Crenshaw, 2002, p. 177).

In other words, different oppressions can meet at a point of intersection and flow along the axes of subordination within a single individual. For example, a black woman suffers the effects of both sexism and racism: not separately, but together, in a mutual affectation.

Digital racism can be analyzed as a new marker of intersectionality since it acts as a form of social exclusion considering the contemporary context intrinsically linked to communication and information technologies. Discussing plural and collective solutions to this problem without addressing the intersectional topic seems unfeasible. By seeking to reclaim what has been denied by coloniality, these paths call us to look at the various markers of oppression that act on subjects and originate from the power axes imposed since colonization.

## 5 Final considerations

In the final part of this essay, we revisit the proposed objective: to discuss digital racism from the perspective of social classification and the coloniality of Being in the context of decolonial criticism, seeking to contribute to discussions raised not only by researchers but by subjects who tell us about digital exclusion based on their own experiences. We attempt to answer these questions from the discussion held throughout the work. We approach digital racism from the perspective of social classification and the coloniality of Being, pointing out that racist practices are legacies of the nations’ colonization and the supremacy of Eurocentric ideals.

On the basis of these discussions, we assert that digital racism is a “new” process of social exclusion intertwined with coloniality, which allows this phenomenon to be analyzed as a marker of intersectionality. The choice for the theoretical essay involves the defense we make, observing the daily scenario of the authors in readings, lives and experiences organized in the woven arguments. In the history of this writing, we understand that we are dealing with concerns that are not yet widely present on the scientific agenda, at least in psychology, with the problematization of digital racism within the horizon of social exclusion. However, a theoretical essay implies an exercise, an invitation to debate, criticism, and collective elaborations. In other words, the theme of digital racism as a facet of social exclusion still needs to be discussed, expanded and reflected upon. The text produced here is an initial and unfinished reflection and invites other researchers, with future research, to dialog on the issue.

Moreover, this essay seeks to point out possible solutions and paths that lead to decoloniality and, consequently, to understanding and confronting racism and digital exclusion. According to Fernández et al. (2021), coloniality is still alive because the epistemologies born from colonial conditions do not allow the roots and routes toward the detachment of these perspectives to be traced - in theory, research and practice. In this sense, we are located in a context of coloniality and colonial logic, even when we value a critical professional practice positioned from a decolonizing point of view that seeks to change these power dynamics. In dialog with Lugones (2003) and Fernández et al. (2021) point out that decoloniality and decolonization are continuous processes of disrupting and interrogating ourselves, our relationships with people and lands, and our understandings of the worlds we traverse. We speak from this place, Latin America, Brazil, a former Portuguese colony, in the process of (re)encountering ourselves, our history, in memory, in re-editing ourselves, through the reflection that decolonial thinking enhances, by activating the reminiscences of the multiple possibilities of being and existing that live within us.

Thus, perspectives that seek to problematize socially instituted asymmetrical relations and reflect our practices in the context of coloniality indicate a procedural movement of continuous disruption and continuous questioning concerning our reality. Based on these reflections, we point to relational Psychology, which is situated and committed to affirming being and its differences, as a way of understanding the subjective unfolding of this exclusionary process and raising spaces for the affirmation of being in its relationship with the world.

## Data Availability

The original contributions presented in the study are included in the article/supplementary material, further inquiries can be directed to the corresponding authors.

## Footnotes

^1^Hackathon is a programming marathon. The term comes from the combination of the English words “hack” (to program in an exceptional way) and “marathon.” It is an event that brings together programmers, designers and other professionals linked to software development with the aim of developing software or a technological solution that meets a specific purpose (Lourenço, 2024).

^2^Between approaches to difference, the understanding adopted in this writing refers to the colonial difference, mobilized by colonialities, which define equality and difference in terms of hierarchical social classification, which justifies and legitimizes epistemic violence.

^3^Although the geographical reference is associated, the advancement of the discussion is understood when we understand that the reference to the margin is included, in the ‘center-periphery’ dimension, of those who are marginalized, in a social process that classifies and, subsequently, hierarchizes - subalternized.

^4^Acronym for Lesbian, Gay, Bisexual, Transgender, *Queer*, Intersex, Asexual and others.

^5^Assuming the decolonial and not the descolonial walks in the affirmation of processuality, given the understanding that tension, as conflict, and also suffering, is part of the subjectivation process of identity-identification, understood in the light of colonialities.

^6^Available at: https://www.cnn.com/shows/united-shades-of-america. The title is a pun on the name of the country, exchanging the word “states” for “shades.”
